# A loss of *Pdxk* model of Parkinson disease in Drosophila can be suppressed by *Buffy*

**DOI:** 10.1186/s13104-017-2526-8

**Published:** 2017-06-12

**Authors:** P. Githure M’Angale, Brian E. Staveley

**Affiliations:** Department of Biology, Memorial University of Newfoundland, St. John’s, NL A1B 3X9 Canada

**Keywords:** Dopaminergic neurons, Drosophila, Parkinson disease, Pyridoxal kinase, Pyridoxal 5′ phosphate, Buffy

## Abstract

**Background:**

The identification of a DNA variant in *pyridoxal kinase* (*Pdxk*) associated with increased risk to Parkinson disease (PD) gene led us to study the inhibition of this gene in the *Dopa decarboxylase* (*Ddc*)-expressing neurons of the well-studied model organism *Drosophila melanogaster*. The multitude of biological functions attributable to the vitamers catalysed by this kinase reveal an overabundance of possible links to PD, that include dopamine synthesis, antioxidant activity and mitochondrial function. Drosophila possesses a single homologue of *Pdxk* and we used RNA interference to inhibit the activity of this kinase in the *Ddc*-*Gal4*-expressing neurons. We further investigated any association between this enhanced disease risk gene with the established PD model induced by expression of *α*-*synuclein* in the same neurons. We relied on the pro-survival functions of Buffy, an anti-apoptotic *Bcl*-*2* homologue, to rescue the Pdxk-induced phenotypes.

**Results:**

To drive the expression of *Pdxk* RNA interference in DA neurons of Drosophila, we used *Ddc*-*Gal4* which drives expression in both dopaminergic and serotonergic neurons, to result in decreased longevity and compromised climbing ability, phenotypes that are strongly associated with Drosophila models of PD. The inhibition of *Pdxk* in the *α*-*synuclein*-induced Drosophila model of PD did not alter longevity and climbing ability of these flies. It has been previously shown that deficiency in vitamers lead to mitochondrial dysfunction and neuronal decay, therefore, co-expression of *Pdxk*-*RNAi* with the sole pro-survival *Bcl*-*2* homologue *Buffy* in the *Ddc*-*Gal4*-expressing neurons, resulted in increased survival and a restored climbing ability. In a similar manner, when we inhibited *Pdxk* in the developing eye using *GMR*-*Gal4*, we found that there was a decrease in the number of ommatidia and the disruption of the ommatidial array was more pronounced. When *Pdxk* was inhibited with the *α*-*synuclein*-induced developmental eye defects, the eye phenotypes were unaltered. Interestingly co-expression with *Buffy* restored ommatidia number and decreased the severity of disruption of the ommatidial array.

**Conclusions:**

Though *Pdxk* is not a confirmed Parkinson disease gene, the inhibition of this kinase recapitulated the PD-like symptoms of decreased lifespan and loss of locomotor function, possibly producing a new model of PD.

## Background

Parkinson disease (PD), the second most common human neurodegenerative disorder, after Alzheimer disease, afflicts about 1% of the population over the age of 50 years of age [1]. The clinical disorders associated with PD are a variable combination of bradykinesia, postural instability, tremor, and rigidity, with a typical positive response to Levodopa [2]. Non-motor symptoms that include autonomic dysfunction, cognitive decline and psychiatric problems can also present [3]. These end-points are mainly attributed to the loss of dopaminergic (DA) neurons of the *substantia nigra* with the degeneration of the nigrostriatal dopaminergic system. However, the neuropathology of PD is known to be more widespread, with many non-dopaminergic nuclei affected, including the locus coeruleus, the brain stem, raphe nucleus, dorsal motor nucleus of the vagus, basal nucleus of the Meynert, amygdala, and hippocampus [4]. PD is characterized by the presence of neuronal inclusions composed of abnormal α-synuclein and generally referred to as Lewy-related pathology [2, 5]. This atypical protein accumulation is believed to lead to cellular toxicity and, eventually, the PD pathogenesis. A majority of PD cases are idiopathic but the emergence of familial cases led to the identification and study of genes that are highly associated with PD [6, 7]. The understanding and exploitation of the genetic basis of PD has revealed over 20 genes that are implicated in PD pathogenesis [8], and highlighted the complexity of this neurodegenerative disease.

The link between vitamin B_6_ and PD incidence has been explored for years, with some studies associating dietary vitamin B_6_ with reduced effectiveness of Levodopa [9]. Other studies have shown the advantages of a higher dietary vitamin B_6_ and the reduced risk of PD [10] or reported low dietary intake of vitamin B_6_ with increased risk to PD [11], either via its antioxidant abilities or through dopamine biosynthesis. Vitamin B_6_ is comprised of three pyridine derivatives or vitamers—which are chemical compounds that have a similar molecular structure and possess similar vitamin activity—known as pyridoxine (PN), pyridoxamine (PM), pyridoxal (PL) and their phosphorylated products pyridoxine-5′-phosphate (PNP), pyridoxamine-5′-phosphate (PMP) and pyridoxal-5′-phosphate (PLP) [12, 13]. PLP is the most metabolically active form and responsible for more than 100 enzymatic reactions [12], predominantly in amino acid metabolism, and is implicated in nervous system function (neurotransmitter synthesis), red blood cell formation (heme biosynthesis), vitamin formation, one-carbon metabolism (nucleic acid synthesis) and as a potent antioxidant [14]. In neuronal function, PLP plays a key role in the metabolism of neurotransmitters, including dopamine, serotonin, glycine, GABA, glutamate, d-serine and histamine [12]. The deficiency of vitamin B_6_ has been implicated in increased risk of cancer, neural decay and accelerated ageing. Mitochondrial oxidative decay is a major contributor to ageing [15, 16]. Mitochondrial function is more dependent on PLP than any other organelle as PLP function as a coenzyme for transaminases that are involved in the catabolism of all amino acids by the urea cycle of the mitochondria [16]. PLP is involved in diverse biochemically important roles in the mitochondria including maintaining energy pathways, homocysteine and glutathione (an antioxidant) biosynthesis. The heme biosynthesis occurs predominantly in the mitochondria and depends on PLP as a coenzyme. The inadequate synthesis of heme can cause mitochondrial decay and oxidative DNA damage [15], whereas its inhibition can cause oxidant leakage, that increases cellular endogenous ROS formation. Vitamin B_6_ has a direct antioxidant activity by preventing superoxide radical formation, glycated haemoglobin formation and erythrocyte lipid peroxidation [17]. The inter-conversion of the pyridines to the biologically active phosphate derivative PLP require the action of pyridoxal kinase, thus, the activation of vitamin B_6_ to its active form, PLP, requires pyridoxal kinase.

Pyridoxal kinase (Pdxk) belongs to the phosphotransferase family of proteins that are involved in the phosphorylation of vitamin B_6_ to pyridoxal-5-phosphate an important co-factor in intermediary metabolism [18, 19]. They contain a ribokinase/pyridoxal domain and are highly conserved, being found in yeast, plants and animals. The association of the gene coding for *Pdxk* with Parkinson disease was through whole-genome expression profiling of human DA neurons, combined with association analysis in differentially regulated genes [20]. A DNA variant, single nucleotide polymorphism, in the *Pdxk* gene has been associated with an increased risk to PD [20], though other studies ruled out the association of the variant *rs2010795* with PD in a cohort of patients [21]. The study did not rule out the existence of the Pdxk variants that may increase the risk for PD. The development of model systems to study potential therapies is important, and as such *Drosophila melanogaster* is a good model organism to study the pathophysiology of movement disorders [22]. The first Drosophila model of PD relied on the expression of human *α*-*synuclein*—which has no known homologue in Drosophila—to induce the PD-like symptoms [23]. Its success anchors on the ability to recapitulate features of human PD such as (1) age-dependent loss in locomotor function (2) LB-like inclusions and (3) age-dependent loss of DA neurons; and is widely used in the study of *α*-*synuclein*-induced neurodegeneration [22–27]. The utilization of the UAS/GAL4 spatio-temporal expression system [28], and the availability of a plethora of promoters or enhancers of which *TH*-*Gal4*, *elav*-*Gal4* and *Ddc*-*Gal4* are employed in modelling PD in flies, makes Drosophila a powerful model organism [22–27]. We have previously shown the pro-survival advantages of *Buffy*, the sole anti-apoptotic *Bcl*-*2* homologue in Drosophila by its rescue of the *α*-*synuclein*-induced phenotypes [29], in the HtrA2 model of PD [30], we extended this study to investigate whether *Buffy* would rescue the loss of *Pdxk*-induced phenotypes in Drosophila.

## Results

### *Pdxk* is evolutionarily conserved across diverse species

**Fig. 1:**
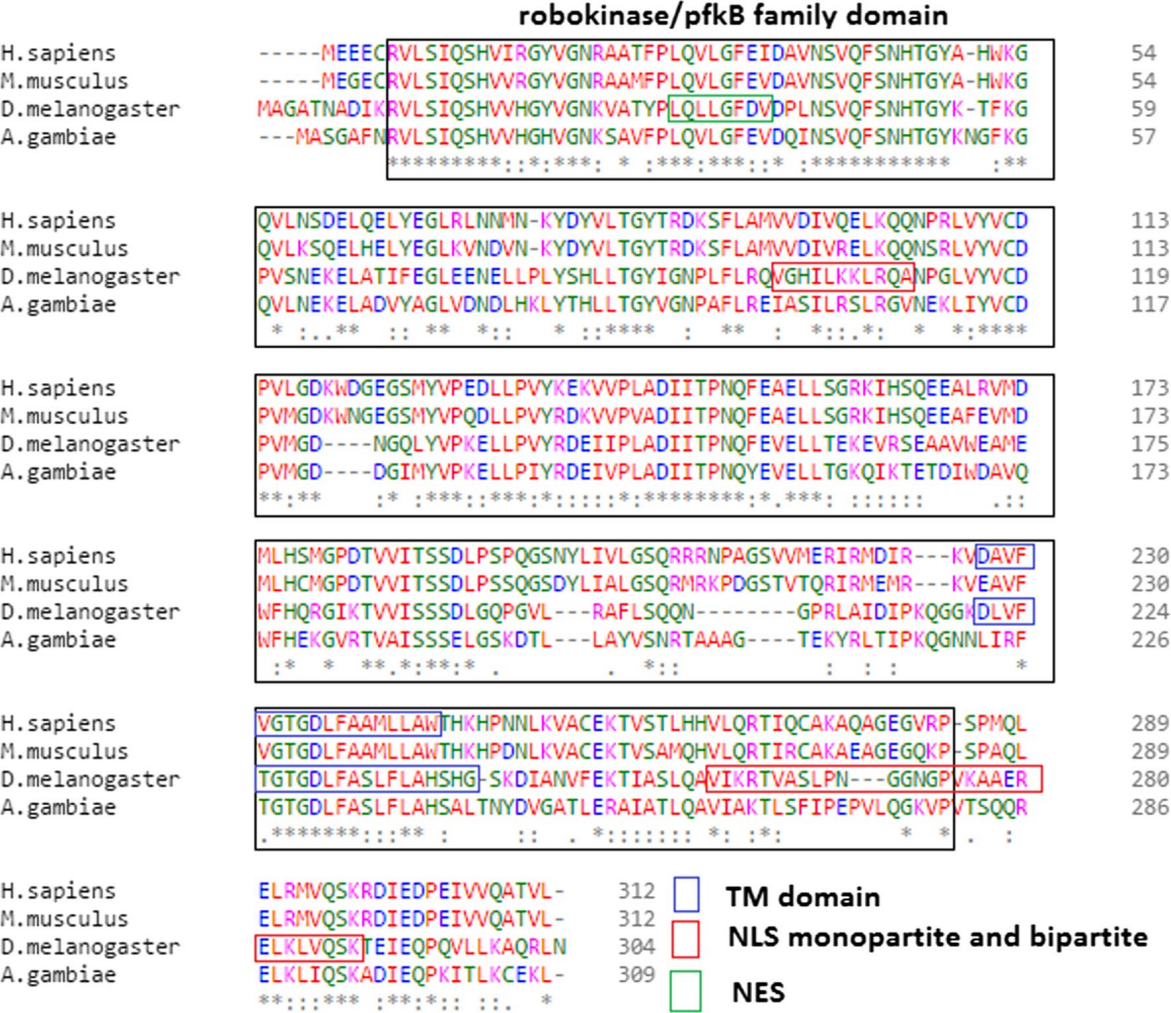
The ribokinase/pyridoxal domain is evolutionarily conserved. A Clustal Omega multiple sequence alignment [33] of *Drosophila melanogaster* Pdxk with that of mammalian and insect homologues shows an evolutionarily conserved kinase domain (*H. sapiens* is *Homo sapiens* NP_003672.1, *M. musculus* is *Mus musculus* NP_742146.1, *A. gambiae* is *Anopheles gambiae* XP_315959.4, and *D. melanogaster* is *Drosophila melanogaster* NP_996031.1). The Drosophila transcript shows presence of motifs for NLS, a weak NES, MTS, and TM domains. Domains were identified using the NCBI Conserved Domain Database (CDD) [31] and the Eukaryotic Linear Motif resource [32]. *Asterisk* indicate the residues that are identical, *colon* indicate the conserved substitutions, *dot* indicate the semi-conserved substitutions. *Colours* show the chemical nature of amino acids. *Red* is small hydrophobic (including aromatic), *blue* is acidic, *magenta* is basic, and *green* is basic with hydroxyl or amine groups

The bioinformatic analysis of protein sequences encoded by the human, mouse, mosquito and fruit fly *Pdxk* gene revealed a highly conserved ribokinase/pyridoxal kinase domain (Fig. 1) as determined by NCBI Conserved Domain Database (CDD) [31] and Eukaryotic linear motif (ELM) resource [32]. Comparison of the human and the Drosophila homologues by NCBI’s BLAST revealed a 46% identity and 64% similarity along the protein sequences. The alignment of protein sequences using Clustal Omega multiple sequence alignment [33] demonstrates the high level of conservation of the kinase domain. The predicted Drosophila protein appears to be localised in the cytoplasm as predicted by MultiLoc2 [34], and to possess active sites and motifs that include a weak nuclear export signal (NES) as determined by NetNES [35], a nuclear localization signal (NLS) that was detected by cNLS Mapper [36], and a transmembrane (TM) domain as predicted by TMpred [37]. Motifs identified by ELM that may contribute to the function of the Drosophila homologue are ubiquitination site, protein phosphatase 1 interacting motif, IAP binding motif, and an Src Homology 2 (SH2) domain binding motif.

### Loss of *Pdxk* decreases lifespan and locomotor ability

**Fig. 2:**
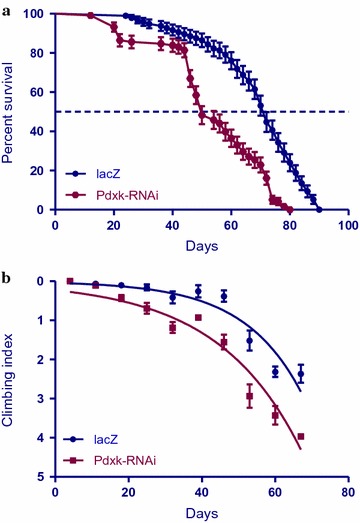
Inhibiting *Pdxk* activity in *Ddc*-*Gal4*-expressing neurons decreases lifespan and impairs locomotor function. **a** The directed expression of *Pdxk*-*RNAi* in the *Ddc*-*Gal4*-expressing neurons using the *Ddc*-*Gal4* transgene results in a decrease in median lifespan when compared to control flies expressing *UAS*-*lacZ*. The genotypes are *UAS*-*lacZ/Ddc*-*Gal4* and *Pdxk*-*RNAi/Ddc*-*Gal4.* Longevity is shown as percent survival [P < 0.05, determined by the log-rank (Mantel–Cox) test and *n* ≥ 200]. **b** The inhibition of *Pdxk* in the *Ddc*-*Gal4*-expressing neurons resulted in a significant decrease in climbing ability as determined by nonlinear fitting of the climbing curves and comparing 95% CI. The genotypes are *UAS*-*lacZ/Ddc*-*Gal4* and *Pdxk*-*RNAi/Ddc*-*Gal4. Error bars* indicate SEM and *n* = 50

The inhibition of *Pdxk* in the *Ddc*-*Gal4*-expressing neurons by RNA interference results in flies with a decreased lifespan and impaired locomotor function. The median lifespan for *Pdxk*-*RNAi* flies was determined to be 50 days compared to 70 days for the control flies that express the *lacZ* transgene (Fig. 2a). When *Pdxk* was suppressed in the *Ddc*-*Gal4*-expressing neurons the flies showed impaired locomotor ability as determined by the nonlinear fitting of the climbing curves (Fig. 2b). The 95% confidence intervals (CI) were 0.038–0.049 compared to 0.051–0.076 for the controls. Taken together, these results indicate an important role for this kinase in the *Ddc*-*Gal4*-expressing neurons of Drosophila as interference with its activity phenocopies some of the well-established phenotypes observed in other Drosophila models of PD.

### Inhibiting *Pdxk* does not suppress *α*-*synuclein*-induced phenotypes

**Fig. 3:**
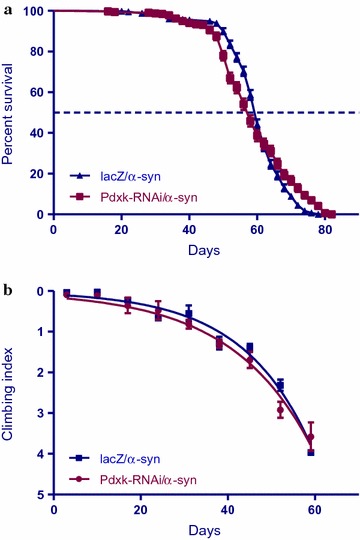
Inhibition of *Pdxk* in the *α*-*synuclein*-induced PD model does not alter phenotypes. **a** The inhibition of Pdxk with *α*-*synuclein* in the *Ddc*-*Gal4*-expressing neurons showed no significant change in lifespan when compared to the control. Genotypes are *UAS*-*α*-*synuclein; Ddc*-*Gal4/UAS*-*lacZ* and *UAS*-*α*-*synuclein; Ddc*-*Gal4/Pdxk*-*RNAi.* Longevity is shown as percent survival [P < 0.05, determined by log-rank (Mantel–Cox) test with *n* ≤ 200]. **b** The co-expression of *Pdxk*-*RNAi* in the *α*-*synuclein* model of PD did not result in any significant age-dependent loss in climbing ability compared to the control. The genotypes are *UAS*-*α*-*synuclein; Ddc*-*Gal4/UAS*-*lacZ* and *UAS*-*α*-*synuclein; Ddc*-*Gal4/Pdxk*-*RNAi.* Analysis was done by nonlinear fitting of the climbing curves and significance was determined by comparing the 95% confidence intervals. *Error bars* indicate SEM and *n* = 50

The co-expression of *Pdxk*-*RNAi* with the expression of *α*-*synuclein* does not alter the diminished longevity nor does it change the observed loss of climbing ability over time. The median lifespan was 58 days for *Pdxk* flies compared to 60 days for control flies (Fig. 3a). A comparison of the climbing curves at 95% CI for Pdxk-RNAi flies was 0.033–0.045 compared to 0.036–0.048 for the controls. This comparison showed that they were not significantly different from each other (Fig. 3b). This implies that the inhibition of *Pdxk* in the *Ddc*-*Gal4*-expressing neurons does not influence the neurotoxic effects of α-synuclein.

### The pro-survival *Buffy* suppresses the loss of *Pdxk* phenotypes

**Fig. 4:**
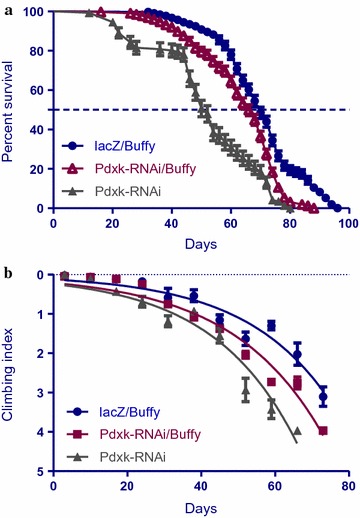
The co-expression of *Pdxk*-*RNAi* with *Buffy* suppresses the *Pdxk*-induced phenotypes. **a** The co-expression of *Buffy* with *Pdxk*-*RNAi* result in the suppression of the observed phenotype of decreased survival. Genotypes are *Ddc*-*Gal4 UAS*-*Buffy/UAS*-*lacZ* and *Ddc*-*Gal4 UAS*-*Buffy/Pdxk*-*RNAi.* Longevity is shown as percent survival [P < 0.05, determined by log-rank (Mantel–Cox) test with *n* ≤ 200]. **b** The co-expression of *Pdxk*-*RNAi* with *Buffy* in the *Ddc*-*Gal4*-expressing neurons results in the suppression of the age-dependent loss in climbing ability. The genotypes are *Ddc*-*Gal4 UAS*-*Buffy/UAS*-*lacZ* and *Ddc*-*Gal4 UAS*-*Buffy/Pdxk*-*RNAi.* Analysis was done by nonlinear fitting of the climbing curves and significance was determined by comparing the 95% confidence interval. *Error bars* indicate SEM and *n* = 50

The co-expression of the pro-survival *Bcl*-*2* homologue *Buffy* with *Pdxk*-*RNAi* in *Ddc*-*Gal4*-expressing neurons results in a slightly increased lifespan and an improved climbing ability. The median survival of *Pdxk*-*RNAi* flies with co-expression of *Buffy* was 68 days when compared to 74 days for the controls that expressed the *lacZ* transgene and *Buffy*, as determined by Log-rank at a P < 0.0001 (Fig. 4a). The climbing ability was significantly improved as determined by a nonlinear fitting of the climbing curves and compared at 95% CI (Fig. 4b). These results suggest a pro-survival role for *Buffy* as exhibited by significant increases in both survival and locomotor function when *Pdxk* is inhibited in the *Ddc*-*Gal4*-expressing neurons.

### Inhibiting *Pdxk* in the developing eye results in disruption of the ommatidial array that can be suppressed by *Buffy*

**Fig. 5:**
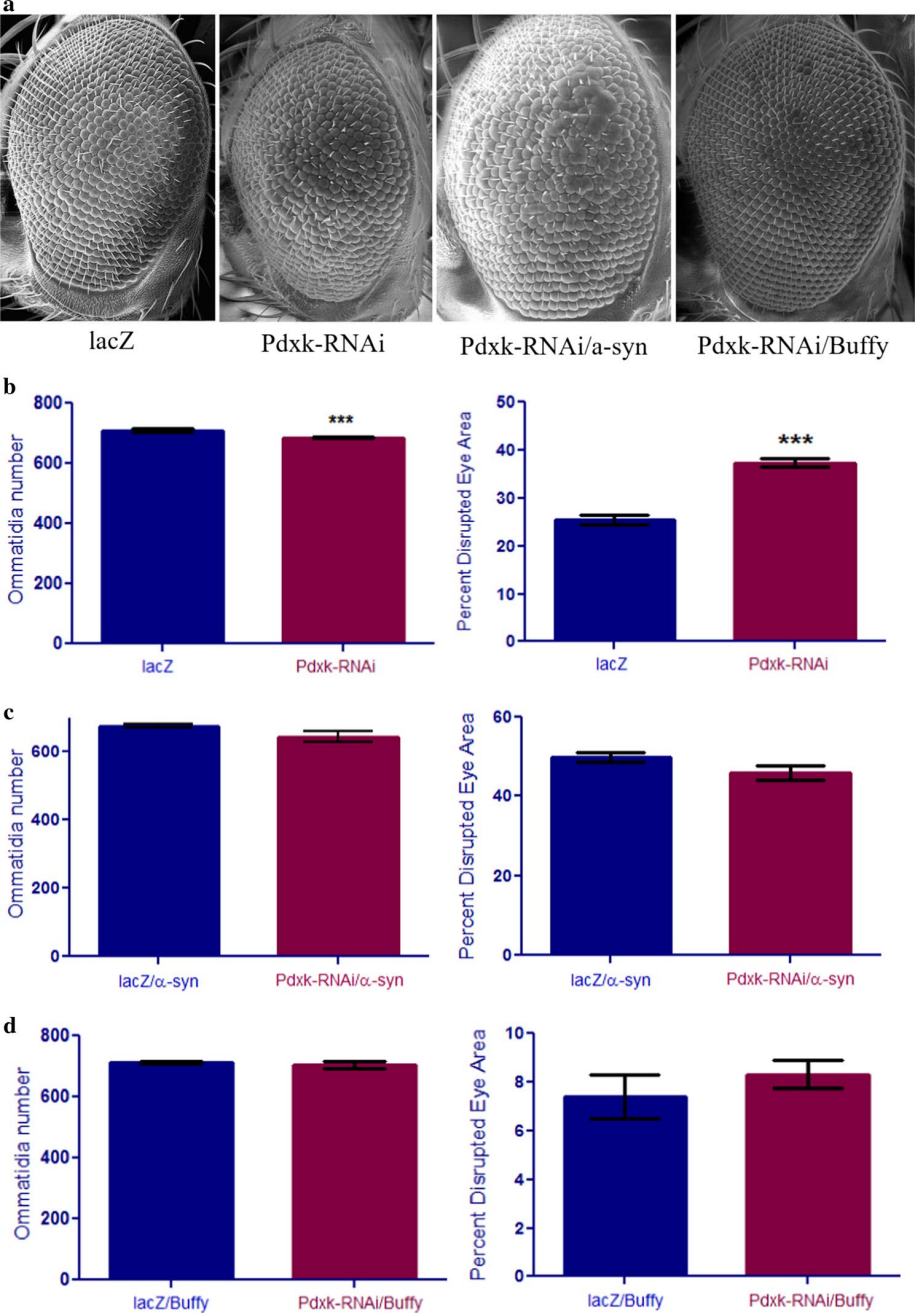
The conditional expression of *Pdxk* in the Drosophila eye results in reduced ommatidia number and increased disruption of the eyes and is suppressed upon co-expression with *Buffy.* **a** Scanning electron micrographs when *Pdxk* is inhibited in the eye (I) *GMR*-*Gal4/UAS*-*lacZ* and (II) *GMR*-*Gal4/Pdxk*-*RNAi*. **b** The biometric analysis of the eyes indicated a slight decrease in mean ommatidia number and a higher percent disruption of the eye when compared to the control. **c** The co-expression of *Pdxk*-*RNAi* with *α*-*synuclein*-expression (I) *UAS*-*α*-*synuclein; GMR*-*Gal4/UAS*-*lacZ* and (II) *UAS*-*α*-*synuclein; GMR*-*Gal4/Pdxk*-*RNAi.* Biometric analysis of *α*-*synuclein*-expression and *Pdxk* inhibition in the developing eye revealed no significance in the number of ommatidia and the degree of ommatidial disruption. **d** Co-expression of *Buffy* with *Pdxk*-*RNAi* (I) *UAS*-*Buffy; GMR*-*Gal4/UAS*-*lacZ* and (II) *UAS*-*Buffy; GMR*-*Gal4/Pdxk*-*RNAi.* Biometric analysis showed restoration of the mean number of ommatidia and ommatidial disruption to control levels. Comparisons were determined by unpaired two-tailed T test (P < 0.05), *error bars* are SEM, *n* = 10 and *asterisks* represent statistical significance

The inhibition of *Pdxk* in the developing eye directed by the *GMR*-*Gal4* transgene results in eyes with a significantly lower mean number of ommatidia, at 686.4 for *Pdxk*-*RNAi* flies compared to 706.9 for the *lacZ* control flies (Fig. 5a, b) as determined by an unpaired T test with a P value of 0.0007. More disruption of the ommatidial array was observed in *Pdxk*-*RNAi* flies, at 37.3% when compared to the *lacZ* control flies at 25.5% (Fig. 5a, b) as determined by an unpaired T test with a P value of 0.0001. The inhibition of *Pdxk* along with *α*-*synuclein* expression did not result in marked differences in either the number of ommatidia or the disruption of the eye between the flies that express *Pdxk*-*RNAi* along with *α*-*synuclein,* when compared to control flies that expressed *lacZ* along with *α*-*synuclein* (Fig. 5a, c). The mean number of ommatidia for *Pdxk*-*RNAi*/*α*-*synuclein* flies was 643.8 compared to 675.6 for the *lacZ/α*-*synuclein* flies, a not significant difference as determined by an unpaired T test (P = 0.0616). Additionally, the disruption of the ommatidial array was not significantly different, at 45.8 and 49.8% respectively as determined by an unpaired T test (P = 0.3117). However, a one-way analysis of variance between *Pdxk*-*RNAi*/*α*-*synuclein* and *Pdxk*-*RNAi* flies revealed that the expression of *α*-*synuclein* along with *Pdxk*-*RNAi* had a significantly severe phenotype in the eye compared to the *Pdxk*-*RNAi* alone flies (Fig. 5a, c), marked by decreased mean ommatidia number and higher disruption of the ommatidial array. It appears that the inhibition of *Pdxk* in the developing eye along with *α*-*synuclein* expression enhances the *Pdxk*-*RNAi*-induced eye phenotype. When we co-expressed *Pdxk*-*RNAi* with *Buffy*, the number of ommatidia for *Pdxk*-*RNAi/Buffy* flies was 704.3 compared to 711.9 for the flies that expressed *lacZ* along with *Buffy* overexpression, this restored the *Pdxk*-*RNAi*-induced eye phenotypes to control levels. Similarly, the disruption of the ommatidial array was down to 8.4% for *Pdxk*-*RNAi/Buffy* flies compared to 6.4% for *lacZ/Buffy* flies. These comparisons were not significant as indicated by an unpaired T test with P values of 0.469 and 0.4115 respectively (Fig. 5a, d). However, a one-way analysis of variance between *Pdxk*-*RNAi/Buffy* and *Pdxk*-*RNAi* alone flies (Fig. 5a, b) showed a remarkable recovery of the *Pdxk*-*RNAi*-induced phenotypes. Taken together, these results suggest that *Buffy* suppresses the developmental eye defects resulting from the inhibition of *Pdxk* in the developing eye.

## Discussion

The directed inhibition of *Pdxk* in the *Ddc*-*Gal4*-expressing neurons of *D. melanogaster* result in decreased lifespan and an age-dependent loss in climbing ability, phenotypes strongly associated with models of PD. Pyridoxal kinase is involved in the conversion of pyridoxal into pyridoxal-5′-phosphate (PLP), an important enzyme cofactor in intermediary metabolism [14]. These B_6_ vitamers seem to be involved in all vital cellular functions, from glucose metabolism to nucleic acid synthesis to being potent antioxidants. Our inhibition of this protein kinase in the *Ddc*-*Gal4*-expressing neurons resulted in shortened lifespan and impaired climbing ability, while in supportive experiments, its inhibition in the developing eye resulted in reduced ommatidia number and a high degree of ommatidial disarray. The involvement of PLP in the conversion of dopa to dopamine is an important step in its synthesis, it seems therefore, that decreased neuronal PLP can mimic the effects of decreased dopamine, a key step in PD development, and possibly of major impact in dopamine-containing cells. It is not surprising therefore, that decrease in the availability of such an important coenzyme by inhibition of the kinase function involved in its conversion results in defective neuronal function that may lead to neurodegeneration.

The expression of *α*-*synuclein* in *Ddc*-*Gal4*-expressing neurons can result in PD-like characteristics in Drosophila [23]. We investigated the possible existence of a link between *α*-*synuclein*-induced phenotypes and the inhibition of *Pdxk* in the *Ddc*-*Gal4*-expressing neurons of Drosophila. Our results indicate that loss of *Pdxk* function in *Ddc*-*Gal4*-expressing neurons that were additionally expressing *α*-*synuclein* did not enhance the *α*-*synuclein*-induced phenotypes of decreased lifespan and impaired climbing ability. Similarly, when we conducted similar comparisons in the neuron rich Drosophila eye, we observed that inhibition of *Pdxk* did not alter the consequences of *α*-*synuclein* expression in the developing eye. Therefore, the toxic effects of α-synuclein are sufficient to generate phenotypes but inhibition of *Pdxk* activity does not confer any additional disadvantage. Alternatively, loss-of-*Pdxk*-induced toxicity may precede the effects of α-synuclein accumulation with the expression of *α*-*synuclein* enhancing the *Pdxk*-*RNAi*-induced phenotypes and specifically in the developing eye. This observation indicates a cell specific mechanism, where the expression of *α*-*synuclein* in the *Pdxk*-*RNAi* background in the developing eye and not in the DA neurons enhances the phenotypes. Additionally, the multitude of biological functions that are dependent on PLP from a dysfunctional antioxidant activity to mitochondrial dysfunction may induce neurotoxicity in these sensitive neurons.

The directed expression of the pro-survival *Bcl*-*2* homologue *Buffy* with *Pdxk* RNA interference resulted in the suppression of the loss of *Pdxk*-induced phenotypes of decreased survival and impaired locomotor ability. Buffy like many other pro-survival Bcl-2 proteins, is thought to be a “guardian” of the mitochondria and confers survival advantages by restricting death-promoting molecules [38, 39]. *Buffy* suppressed the *Pdxk*-induced phenotypes of reduced lifespan and locomotor ability and further restored ommatidia number and decreased the disruption of the ommatidial array. Not only do PLP deficiencies accelerate mitochondrial decay, but they increase ROS radicals [15, 16], a property that make neurons more vulnerable. It is possible that Buffy restores this balance by initiating or participation in pro-survival signals.

## Conclusions

Although *Pdxk* has not been confirmed to be a PD-causative gene, it has been associated to increased risk of the disease. The inhibition of this gene activity by the directed expression of an RNAi transgene in the *Ddc*-*Gal4*-expressing neurons phenocopies PD-like symptoms in Drosophila, and therefore may represent a novel model of PD. This appear to corroborate other studies that show several mechanisms involved in the aetiology of PD. More studies are required to chart out a pathway for *Pdxk* activity in Drosophila, and importantly to show at a molecular level the changes associated with the loss-of-function of this kinase in the development and function of dopaminergic neurons.

## Methods

### Bioinformatic analysis

The protein sequences for Pyridoxal kinase were obtained from the National Center for Biotechnology Information (NCBI; http://www.ncbi.nlm.nih.gov/protein/) and the domains were identified using the NCBI Conserved Domain Database (CDD; http://www.ncbi.nlm.nih.gov/cdd) [31] and the Eukaryotic Linear Motif resource (ELM; http://elm.eu.org/) [40] which focuses on annotation and detection of eukaryotic linear motifs (ELMs) or short linear motifs (SLiMs). The alignment of sequences was performed with Clustal Omega (http://www.ebi.ac.uk/Tools/msa/clustalo/) [33] to show conservation of the kinase domain in the queried organisms. The prediction of the nuclear export signal (NES) was by NetNES (http://www.cbs.dtu.dk/services/NetNES/) [35]. The nuclear localisation signal (NLS) was predicted with cNLS Mapper [36]. The sub-cellular localisation was performed by MultiLoc2 [34] (https://abi.inf.uni-tuebingen.de/Services/MultiLoc2). Transmembrane domains were identified using TMpred [37], a program based on statistical analysis of TMbase (http://www.ch.embnet.org/software/TMPRED_form.html).

### Drosophila media and culture

Stocks and crosses were maintained on a standard medium prepared from cornmeal, molasses, yeast, agar, water and treated with propionic acid and methylparaben to inhibit fungal growth. Stocks were maintained at 23 ± 2 °C while crosses and experiments were carried out at 25 and 29 °C.

### Drosophila stocks and derivative lines

*UAS*-*Buffy* [38] was generously supplied by Dr. Leonie Quinn (University of Melbourne), *UAS*-*α*-*synuclein* [23] by Dr. Mel Feany of Harvard Medical School, and *Ddc*-*Gal4* [41] by Dr. Jay Hirsch (University of Virginia). The *UAS*-*Pdxk*-*RNAi (P{KK108240}VIE*-*260B)* [42] flies were obtained from Vienna Drosophila Resource Center while *GMR*-*Gal4* [43] and *UAS*-*lacZ* flies were obtained from the Bloomington Drosophila Stock Center at Indiana University. The *UAS*-*α*-*synuclein/CyO; Ddc*-*Gal4/TM3; UAS*-*α*-*synuclein/CyO; GMR*-*Gal4; UAS*-*Buffy/CyO; Ddc*-*Gal4;* and *UAS*-*Buffy/CyO; GMR*-*Gal4* complex lines were generated following a standard protocol as previously described [44, 45]. They expressed *α*-*synuclein* or overexpressed *Buffy* either in the *Ddc*-*Gal4*-expressing neurons using the *Ddc*-*Gal4* transgene or in the developing eye using the *GMR*-*Gal4* transgene. Analysis of complex lines was confirmed via PCR reactions and gel electrophoresis.

### Ageing assay

Multiple matings of each genotype were carried out and male flies collected upon eclosion and analysed following a protocol that has been described [29, 46]. At least 200 flies were aged per genotype and scored every 2 days for the presence of deceased adults [47]. Longevity data was analysed using the GraphPad Prism version 5.04 and survival curves were compared using the Log-rank (Mantel–Cox) test with significance determined with a 95% confidence interval, at a P ≤ 0.05 with Bonferroni correction.

### Climbing assay

A batch of male flies was analysed for their ability to climb according to a standard protocol [48]. In brief, every 7 days after eclosion, 50 or fewer males of each genotype were assayed for their ability to climb. Climbing indices were computed and analysed with GraphPad Prism version 5.04. Climbing curves were fitted using non-linear regression and compared using 95% confidence interval with a 0.05 P value.

### Scanning electron microscopy of the Drosophila eye

Male flies were prepared for scanning electron microscopy using a standard protocol as previously described [29]. Ten different eye images for each genotype were analysed using the National Institutes of Health (NIH) ImageJ software [49] and biometric analysis performed using GraphPad Prism version 5.04. The percentage disruption of the eye was calculated as previously described [50]. Statistical analysis comprised of unpaired student T test and one-way analyses of variance with Newman–Keuls multiple comparison post-test and significance determined at P values equal or less than 0.05.

## Abbreviations

DA: dopaminergic
Ddc: dopa decarboxylase
GMR: glass multiple reporter
PD: Parkinson disease
Pdxk: pyridoxal kinase
PLP: pyridoxal-5′-phosphate
RNAi: ribonucleic acid interference
SEM: standard error of the mean
